# Interventions for the unhoused individual with schizophrenia: a civilized plan

**DOI:** 10.1017/S109285292500001X

**Published:** 2025-03-21

**Authors:** Charles L. Scott

**Affiliations:** Department of Psychiatry and Behavioral Sciences, University of California Davis, Sacramento, CA, USA

**Keywords:** unhoused, homelessness, schizophrenia, housing first, harm reduction, psychosis, compulsory treatment order, anosognosia

## Abstract

This article provides an overview of individuals with schizophrenia who become unhoused and explores current approaches to managing this severe illness in those who often do not want care or believe they need it. Individuals with schizophrenia and who are unhoused face numerous adverse consequences including premature mortality and increased rates of suicide. There is a dearth of research evidence demonstrating efficacy of the Housing First (HF) model and harm reduction approach in decreasing psychotic symptoms in individuals with schizophrenia. Ensuring medication adherence in individuals with psychosis, both housed and unhoused, is important to prevent delays in untreated psychosis and chronic deterioration.

## Introduction

Globally, nearly 24 million individuals are afflicted with schizophrenia^1^, a severe and frequently disabling disorder vastly overrepresented among the homeless population. Schizophrenia involves a loss of touch with reality, with symptoms characterized by hallucinations, delusions (fixed false beliefs), disorganized speech and behavior, diminished emotional expression, and a loss of motivation.^2^ Coupled with the presence of these devastating symptoms, individuals with schizophrenia are often unaware that they have this disease, a phenomena known as “anosognosia.”^3^The combination of having a psychotic illness while being unaware of the illness makes interventions for unhoused individuals with schizophrenia particularly challenging. The faces and deteriorating lives of this population are increasingly impossible to ignore. As one walks down the street of a major urban city, seeing a disheveled and often half-naked human screaming in agony at voices that do not exist and running in fear from unfounded foes, society is faced with an obvious moral question: Is this the best we can do for those most in need?

This article provides an overview of individuals with schizophrenia who are unhoused and explores current approaches to managing this severe illness in those who often do not want care or believe they need it. For purposes of this article, the terms “homelessness” and “unhoused” are used interchangeably.

**Homelessness definitions**

When reviewing the prevalence of homelessness in any community or country, one must first consider the definition of homelessness used to calculate this statistic. In the United States, the Department of Housing and Urban Development (HUD) defines “literally homeless” as an “individual or family who lacks a fixed, regular, and adequate nighttime residence” as exemplified by one of the following 3 categories^4^:

1. The individual resides at night in a private or public place that is not meant for human habitation. This situation is often referred to as unsheltered homelessness or “sleeping rough” and includes living on the street, in a vehicle, or in an abandoned building.
2. The individual lives in some type of temporary shelter, such as an emergency or crisis shelter, transitional housing, or safe haven (SH) program. This category is considered “sheltered homelessness.”
3. The individual resides in an institution for 90 days or less and immediately prior to living in the institution experienced either sheltered or unsheltered homelessness. A person who is briefly incarcerated or hospitalized yet did not have a fixed regular nighttime residence prior to institutional placement meets this definition of being homeless.^4^

Some individuals who do not have a fixed regular residence may not be included in the above definition and are referred to as the “hidden homeless.” For example, a person who is “couch surfing” and stays with different friends or family because they do not have their own housing is likely not counted in official homelessness statistics.

Homelessness can also be categorized based on the time frame that the individual is unhoused. According to HUD, chronic homelessness involves an individual with a disability (such as a substance use disorder or mental illness) who has lived in a sheltered or unsheltered living environment for at least 12 months or at least 4 separate occasions during the last 3 years (if the combined occasions are at least 12 months in duration). Transitional homelessness, therefore, involves individuals who do not have housing for less than 12 months. Transitional homelessness is the most common type of homelessness and may result from a major life stressor, such as losing a job, experiencing a change in relationship status, enduring a natural disaster, or having a sudden change in financial circumstances.^4^

### Schizophrenia and homelessness

Individuals with a serious mental illness (SMI) are at a substantial risk of facing homelessness when compared to the general population. Of note, men and women in the United States who are diagnosed with a SMI such as schizophrenia have a risk of becoming unhoused that is 10–20 times greater than the general population.^5^ US veterans diagnosed with schizophrenia are likewise at increased risk of facing homelessness. In their study of 102 207 veterans, Lin et al. (2022)^6^ found that the frequency of homelessness for veterans with schizophrenia was 28.2%, dramatically higher than the frequency of homelessness in a matched cohort of veterans without schizophrenia (7.2%) and even higher when compared to the prevalence of homelessness of 0.2% found in the general population.

Researchers have also studied unhoused individuals to assess the prevalence of schizophrenia or other psychotic disorders in this population. In 2019, Ayano et al.^7^ published a systematic review and meta-analysis describing the prevalence of schizophrenia and other psychotic disorders among homeless people. Their review included 31 studies involving 51 295 unhoused individuals. The authors included studies from both developed and developing countries. Developed countries were described as those with a longstanding market economy and strong research traditions. Developed countries included in this study were the United States of America, Canada, Germany, Spain, France, Scotland, the United Kingdom, Japan, and Australia. Developing countries for purposes of this study included China, Ethiopia, Ireland, and Serbia.

The definition of homelessness used for this review was broad, including people sleeping in public places, living in shelters, or marginal accommodations. Most studies included in this meta-analysis utilized the DSM criteria for diagnosing schizophrenia and other psychotic disorders. The authors found the following prevalence of schizophrenia and other psychotic disorders among homeless people: overall, psychosis—21.21%; schizophrenia—10.29%; schizophreniform disorder—2.48%; schizoaffective disorder—3.53%, and psychotic disorders not otherwise specified—9%. Similar results were noted by Gutwinski et al.^8^, which noted a 12-month prevalence rate of schizophrenia spectrum disorders of 12.4% from their systematic review and meta-analysis of the published literature on this topic.

Barry et al.^9^ conducted a meta-analysis to also assess the current and lifetime prevalence of mental health disorders among people experiencing homelessness age 18 or older. These authors included 85 studies in their final review, which consisted of 48 414 participants (77% male; 22% female). Most studies included in this review were from the United States (n = 36), with studies from Canada (n = 8) and Germany (n = 7) rounding out the top 3. Although the definition of homelessness varied among the studies, the majority (n = 71) included individuals who were either living in a shelter or other places not intended for housing, such as the streets. Findings indicated a current prevalence of mental health disorders of 67% and lifetime prevalence of mental health disorders of 77% among people experiencing homelessness. Although substance use disorder had the highest current prevalence of all disorders (44%) in this population, the current prevalence of schizophrenia and psychotic disorders was 21% and the lifetime prevalence was 25%.

The results of these studies are clear. Both current and lifetime prevalence of schizophrenia/psychotic disorders in the homeless are substantially higher than found in the housed general population. To more clearly illustrate, 1 in 5 unhoused individuals living in shelters or on the streets are suffering from a current psychotic disorder and 1 in 4 have a lifetime history of schizophrenia or a psychotic disorder. This finding contrasts dramatically with the estimated prevalence in the general population, which ranges from 0.3% to 0.7%.^2^ These numbers become even more alarming when reviewing the prevalence of schizophrenia in transitional shelters (whose goals is to assist unhoused person with a SMI). Viron et al.^10^ found that schizophrenia-spectrum disorders were present in 67.6% of this group, far greater than mood disorders present in 35.1% of the sample.

With the high prevalence of psychosis in unhoused individuals, the following question arises: Are those with schizophrenia or other psychotic disorders at risk to move into chronic homelessness, including homeless shelters? Burton et al.^11^ examined this question by comparing homeless men with psychosis who lived in central Melbourne over a 12-month period in 2018 with data related to homeless men with psychosis in 2006. These authors found that the mean time spent without shelter for homeless psychotic men in 2018 (149 days) was over double that in 2016 (72 days). Greater than 40% of the 2018 sample were “sleeping rough.” The findings raise concern that a significant number of unhoused men with psychosis are becoming increasingly entrenched in homeless settings, which results in worsened continuity of care combined with suboptimal treatment of psychosis.

With research confirming that those with schizophrenia have higher rates of experiencing homelessness in their lifetime and that up to 25% of homeless have a schizophrenia spectrum disorder, is there somehow a link between this severe mental illness and homelessness? Several proposed theories may account for why individuals with schizophrenia are at an increased risk for becoming unhoused when compared to the general population. First, many individuals with schizophrenia, particularly when untreated, experience a gradual deterioration that adversely impacts reality testing, cognition, and functioning. A cascade of negative outcomes results in social withdrawal and difficulty maintaining employment with a loss of income necessary to fund housing. Second, persons with schizophrenia have a high comorbidity of alcohol or other substance use disorder, both additional risk factors related to becoming homeless.^12^ Third, with the deinstitutionalization movement that began in the 1960s, the community mental health system did not have the resources to manage a severely mentally ill patient population. Without an appropriate level of intervention, many individuals with schizophrenia failed to receive appropriate medication, monitoring, and follow-up in the community with a subsequent shift of psychiatric inpatient care from hospitals to jails and prisons.^13^

## Impact of homelessness on individuals with schizophrenia

Individuals with schizophrenia experience higher rates of co-occurring medical disorders, substance use disorders, other psychiatric disorders, premature mortality,^12^ ^,^^14^ and suicide when compared to the general population.^15^ For the unhoused person with schizophrenia, these adverse consequences are heightened as they have limited access to medical and mental health treatment. It is no surprise, therefore, that unhoused individuals with schizophrenia face more frequent psychiatric hospital readmission rates, an emotional burden for the individual as well as a financial burden for society. In their retrospective study of 207 patients who had been psychiatrically hospitalized, Lorine et al.^16^ evaluated 207 patients who were discharged and followed up at 3 different time periods to examine readmission rates. The time frames were readmission within 15 days (Group 1), readmission within 3–6 months (Group 2), and not being readmitted from at least 12 months (Group 3). Of this sample, 50% had schizophrenia or schizoaffective disorder and 24% of the sample were homeless. The study found that having a diagnosis of schizophrenia or schizoaffective disorder increased the odds of being readmitted within 15 days versus not being readmitted within 12 months by nearly 18 times. For those with a diagnosis of schizophrenia or schizoaffective disorder, being homeless increased their odds of readmission within 15 days versus not being readmitted by nearly 30 times. These results highlight the particular risk that having both a schizophrenia spectrum disorder and being unhoused plays in hospital readmission.

Unhoused individuals with schizophrenia are also at an increased risk of becoming both perpetrators and victims of violence. A diagnosis of schizophrenia in and of itself confers an increased risk of violence toward others when compared to the general population.^17^ Untreated schizophrenia is characterized by active psychosis, which typically includes symptoms of paranoia, hallucinations, and false beliefs about others and one’s environment. In an analysis of 204 studies examining the relationship between psychopathology and aggression, Douglas et al.^18^ found that psychosis was the most important predictor of violent behavior in an individual. Although significant research indicates that unhoused individuals without schizophrenia have higher rates of violence and criminal offending,^19^ ^,^ ^20^ does the combination of both circumstances heighten the risk of criminal offending further? The answer is yes. Research by Nilsson et al.^21^ indicates that individuals with a severe mental illness (eg, schizophrenia or bipolar disorder) who are unhoused have higher rates of violence than those who are housed.

Unhoused individuals with schizophrenia are at increased risk of being victims of violence in addition to their increased risk of victimizing others. In their study, Roy et al.^22^ reviewed 21 studies investigation the relationships of persons experiencing homelessness with serious mental illness (PEHSMI) to violence and victimization. The authors reviewed 15 studies specific to contacts with the criminal justice system and 6 studies specific to the prevalence of victimization. Their analysis indicated that PEHSMI had lifetime arrest rates ranging from 63% to 90%, lifetime conviction rates ranging from 28% to 80%, lifetime incarceration rates ranging from 48% to 67%, and lifetime victimization rates ranging from 74% to 87%.

## Interventions for unhoused individuals with schizophrenia

With the goal of implementing the most effective treatment approaches for unhoused individuals with schizophrenia or other psychotic disorders, 2 competing models have been implemented: Treatment First versus Housing First (HF). The Treatment First model, also known as the “Continuum of Care” model, was the dominant model for managing psychiatric patients who had been discharged into the community during the deinstitutionalization movement of the 1960s and 1970s. This model typically consists of a linear model of care, where individuals with a psychiatric disorder have a step wise progression of services that help prepare the individual for independent living, a process known as “housing readiness.”^23^

Under the Treatment First model, the unhoused individual is typically expected to demonstrate sobriety and readiness to accept mental health treatment while in temporary housing. In the Treatment First model, case managers evaluate whether the person has sufficient life skills to live without onsite supervision.^24^ Once the individual is deemed “housing ready,” then independent housing is considered. Individuals who refuse to engage in psychiatric treatment, who continue to use substances, have a history of violence or incarceration, or problematic behaviors are not typically placed in independent housing under this model.

In contrast, HF model emphasizes the importance of first being placed in permanent housing regardless of whether the person is actively psychotic, using alcohol or drugs, agrees to occupational rehabilitation, or accepts recommended treatment interventions. The HF model’s origins can be traced to the Consumer Preference Supported Housing (CPSH) model that arose from a private nonprofit social services organization in New York City known as Pathways to Housing, Inc. Under this model, housing is considered a right for all individuals and interventions are client centered. The CPSH model is the precursor for the HF model, and its foundation includes the following tenants^25^:

1. Unhoused individuals with a SMI can successfully live independently in housing of their choice with appropriate supports;
2. The individual chooses their own housing;
3. Housing is rented from a community landlord who does not provide the support services;
4. The individual does not lose their housing when they are in a clinical crisis, such as a substance use relapse or psychotic episode;
5. Services are provided by a community ACT team and available 24 hours a day;
6. The individual selects the type, frequency, and order of services chosen;
7. No form of treatment is required, including sobriety or medication compliance. Instead, the harm reduction model is used to address alcohol and drug abuse.

In their 2004 research examining the longitudinal effects over a 2-year period of a HF program for homeless individuals with severe mental illness, Tsemberis et al.^26^ compared 126 participants assigned to the Continuum of Care model to 99 participants assigned HF model program. Fifty-three percent of those enrolled in the study had a psychotic disorder diagnosis. Although those placed into the HF program were able to maintain their independent housing over the 24-month period, no differences were found in substance use or psychiatric symptoms between HF versus the Continuum of Care participants.

In their systematic review of 72 articles, Aubry et al.^27^ examined the effectiveness of permanent supportive housing and income assistance for homeless individuals in high-income countries. According to these researchers, most of the studies on permanent supportive housing they reviewed included individuals with severe mental illness who had been unhoused. The authors concluded that both permanent supportive housing and income assistance significantly improved housing stability at 6 years of follow-up. However, this systematic review did not provide evidence that unhoused individuals with permanent supportive housing had improvements in their mental health or substance use compared with controls. In other words, HF may increase housing stability but has not been shown to decrease symptoms of SMI. This finding should inform public policy that to decrease the psychiatric disease burden, antipsychotic medications should be combined with HF to improve the mental health outcomes of unhoused individuals with schizophrenia and other forms of active psychosis.

In their subsequent study, Loubiere et al. ^28^ studied the effects of the HF model among homeless people regarding housing stability, quality of life, healthcare use, mental symptoms, and addiction issues. Researchers examined data from a randomized controlled trial involving homeless or precariously housed adults with severe mental illness from 4 French cities. A total of 703 participants were selected for this study, and all had a diagnosis of either bipolar disorder (30%) or schizophrenia (70%) according to the Diagnostic and Statistical Manual of Mental Disorders, 4th edition, text revision (DSM-IV-TR). In addition to having a bipolar disorder or schizophrenia disorder diagnosis, participants had to have at least 1 of the following: 1. two or more psychiatric hospitalizations in the past 5 years; 2. co-occurring alcohol or substance use disorder; or 3. history of arrest or incarceration within the previous 2 years. Participants who could not provide informed consent for the study were excluded.

The study participants were divided into 2 groups for comparison outcomes: a treatment-as-usual (TAU) group (n = 350) and a HF group (n = 353). For those who were assigned to the TAU group, their housing situations varied, with some individuals living on the streets, with friends or families, or in slums. TAU interventions involved preexisting programs and services for the homeless. These services included outreach teams, day-care facilities, access to emergency shelters and transitional shelters, residential facilities with medical accommodations if needed, and independent housing. In contrast, the HF model involved the provision of independent housing with housing subsidies and assertive community treatment provided by a mobile support team. Both groups were followed over a 48-month period. Both the TAU and HF groups improved in measures of recovery although no statistical difference in recovery outcomes was noted. The HF group compared to the TAU group had a lower use of hospital services. However, no significant differences were found between the 2 groups related to self-reported mental symptoms or substance dependence. In fact, HF participants experienced higher alcohol consumption between baseline and 40 months. The findings in this study that HF did not reduce mental health symptoms replicate the results from the Aubry et al.’s^27^ meta-analysis described above.

Whether or not a homeless individual with schizophrenia is housed, does compulsory treatment, most often involuntary medication administration, improve outcome? Compulsory community treatment orders (CTOs) are legally mandated orders that require psychiatric treatment for identified individuals with severe mental illness who do not voluntarily accept treatment and are considered a risk of harm to self or others or are unable to care for themselves. Failure to follow requirements in CTOs may result in involuntary psychiatric hospitalization. In some jurisdictions, CTOs require a showing that the individual lacks medical decision-making capacity regarding the use of psychotropic medications.^29^

In the 2017 Cochrane review of compulsory community treatment (CCT) for people with mental illness, Kisely and Campbell^30^ reviewed 3 randomized controlled clinical trials of CCT compared with standard care for people with SMI. The authors concluded that their review did not demonstrate that those receiving CCT had differences in improvement in the areas of service use, social functioning, or quality of life compared with those who received voluntary care or brief supervised discharge. However, those receiving CCT were less likely to be victims of violent or nonviolent crime.

CTOs are considered controversial with debated pros and cons of their usage. Suggested benefits of CTOS include earlier treatment intervention that helps prevent mental health deterioration, increased involvement of family and monitoring clinicians, decreased recurrent hospitalizations, decreased interaction with the criminal justice system, and decreased victimization. Concerns regarding the use of CTOs include therapeutic alliance disruption, adverse medication side effects, stigmatization, disproportionate application to people of color or indigenous populations, and a resulting reluctance of the patient to seek future treatment.^31^

Perhaps CTOs are more effective for some individuals than others, depending on the diagnosis. For example, Beaglehole et al.^32^ reviewed nearly 15 000 patients in New Zealand who were placed on a CTO over a 10-year period between January 2009 and December 2018. This study examined the number of psychiatric inpatient admissions per year for individuals on CTOs for a range of psychiatric disorders. These researchers found that the use of CTOs for individuals with a psychotic disorder resulted in reduced hospitalization admissions and frequency. In contrast, individuals with dementia, bipolar disorder, major depressive disorder, and personality disorder had more frequent hospital admissions that were also of longer duration. The authors concluded that compulsory treatment for individuals with psychotic disorders appeared effective in reducing psychiatric hospital admissions and, therefore, relevant in decreasing the disease burden of psychosis.

## Optimizing interventions for unhoused individuals with schizophrenia

Unhoused individuals with schizophrenia face an array of challenges, not the least of which is survival. With increased mortality and suicide rates, what are common sense approaches to caring for these persons who may not want and even refuse treatment? First, practitioners and policymakers should be familiar that many persons with schizophrenia suffer from anosognosia, thereby limiting their ability to appreciate their symptoms and robbing their rational capacity to accept or refuse treatment. Research indicates that between 30 and 50% of patients with schizophrenia lack insight as a prevalent feature of their disorder.^33^

When assessing for decisional capacity to accept or refuse treatment, evaluating the individual’s insight into their mental illness may assist in determining whether treatment refusal is linked to an unawareness that they are psychotic and could benefit from treatment. A consensus definition for assessing insight includes addressing the following questions^34^:

1. Is the person aware that they have a mental illness?
2. Does the person understand the need for treatment?
3. Is the person aware of the potential adverse social consequence related to their mental disorder?
4. Is the person aware that they have symptoms?
5. Is the individual able to attribute their symptoms to a mental disorder?

One widely used tool to assess insight in clinical trials and epidemiological studies is the Scale to Assess Unawareness in Mental Disorder (SMUD).^35^ An abbreviated and more practical version of the SUMD has been developed to assist clinical evaluators assess a patient’s insight into their mental illness. In contrast to the full SMUD, which has 74 items, the abbreviated SMUD has only 9 items which are rated on a severity scale. This abbreviated version rates the following 9 items as related to their mental awareness:

1. mental disorder;
2. consequences of a mental disorder;
3. effects of drugs;
4. hallucinatory experiences;
5. delusional ideas;
6. disorganized thoughts;
7. blunted affect;
8. anhedonia; and
9. lack of sociability.

This abbreviated version has demonstrated that it is a valid instrument for measuring insight in patients with schizophrenia and can accurately assess insight in clinical settings.^36^

Second, when balancing liberty interests in refusing effective medication treatments for schizophrenia versus the treatment benefits, the reality that medications decrease psychotic symptoms and improve outcomes should not be ignored. Large meta-analyses have demonstrated that oral antipsychotics effectively decrease acute psychotic symptoms.^37^ Research also indicates that long-acting antipsychotic injectables are efficacious in treating acutely psychotic individuals with schizophrenia-spectrum disorders.^38^ Because many individuals with schizophrenia have poor medication adherence, the use of a long-acting injectable helps optimize the delivery of an effective medication for often debilitating symptoms. Failure to provide antipsychotic treatment as early as possible has been associated with negative outcomes. In their research studying 2 longitudinal cohorts of patients with first-episode psychosis, Drake et al.^39^ found that a long duration of untreated psychosis (DUP) was associated with a reduced treatment response over time. DUP represents the time between onset of the first threshold psychotic episode and the initiation of treatment. Longer DUP has demonstrated numerous negative outcomes including less likelihood of symptoms going into remission along with a decreased quality of life and level of functioning.^40^ When faced with individuals with schizophrenia who refuse treatments that assist in bringing them closer to reality, psychiatrists should be knowledgeable about the negative impact of delaying treatment so that they can meaningfully inform the relevant decision-maker responsible for treatment refusal overrides. As highlighted above, individuals with schizophrenia are at a much greater risk of early death, completed suicides, and becoming unhoused. These adverse consequences of remaining untreated are relevant when considering compulsory medication to maximize positive treatment outcome.

Third, although the HF approach has shown that individuals with a severe mental disorder who are placed into housing have less days homeless, the research has not demonstrated that those with SMI experience actual symptom reduction with HF alone. Although being placed into housing may decrease the stress and trauma of living on the streets, the HF model by itself is not an effective treatment for the psychotic symptoms of schizophrenia. Combining the HF approach with consideration of compulsory antipsychotic treatment in symptomatic treatment refusers is essential to prevent deterioration and maximize functioning. As noted above, compulsory treatment orders for individuals suffering from psychosis have demonstrated their utility in decreasing hospitalizations for individuals experiencing psychosis.

Fourth, intensive case management and assertive community treatment provide meaningful support and assistance for individuals with schizophrenia. However, for some individuals with schizophrenia, this level of care does not adequately manage their needs or symptoms. Lamb and Weinberger^13^ emphasize the need for more 24-hour structured care facilities as part of the community mental health system. This level of community care will assist those individuals whose psychosis is refractory to treatment as well as decrease the risk of diversion into the criminal justice system. Future policymakers and stakeholders should recognize that current long-term care facilities are not the equivalent of the “snake pit” hospitals of the past, whose horrific conditions understandably played a role in deinstitutionalization of the mentally ill. Moving forward, such 24-hour treatment facilities will likely have an important role in the continuum of care for individuals with schizophrenia who are unhoused or at risk for becoming homelessness due to their disability.

## Summary

Individuals with schizophrenia are at an increased risk of becoming chronically homeless. While attempting to survive on the streets or in a shelter, they face an onslaught of challenges, from being personally victimized, diverted into the criminal justice system, developing serious untreated medical complications, and dying prematurely. Being untreated and actively psychotic represents its own form of psychic torture. What “works best” includes the following key principles:

1. A rational balance of the individual with schizophrenia’s liberty interests with restoration of their sanity and dignity through implementation of compulsory treatment orders when indicated.
2. A recognition that the HF approach alone for individuals with active symptoms of schizophrenia does not address the negative long-term medical and societal outcomes of untreated psychosis. More structured interventions with required treatment involvement are necessary as part of the permanent housing approach.
3. The Harm Reduction approach for individuals with schizophrenia and a co-occurring substance use disorder is unlikely to be effective and may actually perpetuate psychosis, particularly with substances such as cannabis, stimulants, and hallucinogens.

Psychiatrists and mental health professionals play a crucial role in educating policymakers, judges, and other stakeholders that effective treatment and medication therapy work. Society does not have to ignore psychotic humans in despair on the street under the guise of respecting them. In the novel “My Several Worlds,” Pearl *S. Buck* may have said it best when she writes “The test of a civilization is in the way that it cares for its helpless members.”^41^ Moving forward, we should better care for members of our society who need our help. We should pass this basic test of civilization.
